# Changes in marital quality over 6 years and its association with cardiovascular disease risk factors in men: findings from the ALSPAC prospective cohort study

**DOI:** 10.1136/jech-2017-209178

**Published:** 2017-10-09

**Authors:** Ian Bennett-Britton, Alison Teyhan, John Macleod, Naveed Sattar, George Davey Smith, Yoav Ben-Shlomo

**Affiliations:** 1 School of Social and Community Medicine, University of Bristol, Bristol, UK; 2 Institute of Cardiovascular and Medical Sciences, University of Glasgow, Glasgow, UK; 3 MRC Integrative Epidemiology Unit, University of Bristol, Bristol, UK

**Keywords:** cohort study, cardiovascular risk factors, marital quality, male health, ALSPAC

## Abstract

**Background:**

Marital relationship quality has been suggested to have independent effects on cardiovascular health outcomes. This study investigates the association between changes in marital relationship quality and cardiovascular disease (CVD) risk factors in men.

**Methods:**

We used data from The Avon Longitudinal Study of Parents and Children, a prospective birth cohort study (Bristol, UK). Our baseline sample was restricted to married study fathers with baseline relationship and covariate data (n=2496). We restricted final analysis (n=620) to those with complete outcome, exposure and covariate data, who were married and confirmed the study child’s father at 6.4 years and 18.8 years after baseline. Relationship quality was measured at baseline and 6.4 years and operationalised as consistently good, improving, deteriorating or consistently poor relationship. We measured CVD risk factors of blood pressure, resting heart rate, body mass index, lipid profile and fasting glucose at 18.8 years after baseline.

**Results:**

Improving relationships were associated with lower levels of low-density lipoprotein (−0.25 mmol/L, 95% CI −0.46 to −0.03) and relative reduction of body mass index (−1.07 kg/m^2^, 95% CI −1.73 to −0.42) compared with consistently good relationships, adjusting for confounders. Weaker associations were found between improving relationships and total cholesterol (−0.24 mmol/L, 95% CI −0.48 to 0.00) and diastolic blood pressure (−2.24 mm Hg, 95% CI −4.59 to +0.11). Deteriorating relationships were associated with worsening diastolic blood pressure (+2.74 mm Hg, 95% CI 0.50 to 4.98).

**Conclusions:**

Improvement and deterioration of longitudinal relationship quality appears associated with respectively positive and negative associations with a range of CVD risk factors.

## Introduction

Recognition of an association between marital status and health outcomes in observational data is longstanding.^1–5^ The extent to which this observed relationship is due to bias by selection into marriage or protective effects of partnership remains debated.^6 7^

Many mechanisms have been proposed to explain how marital quality might confer health benefits,^8^ but ‘supporting’^9 10^ positive behaviours or ‘buffering’^9^ stress are common themes. It follows that longitudinal relationship quality is a more incisive proxy for how ‘supportive’ or ‘stress-buffering’ a relationship is over time, than representations of marital quality at a single point.

Studies of the effects of relationship quality within marriages control for marriage selection, allowing investigation of the protective effects of partnership in isolation.^8^ The majority of studies addressing the effects of marital quality on health have focused on cardiovascular disease (CVD). This is due primarily to the burden of CVD^11^ and our understanding of the role of modifiable risk factors.^12^

Most studies of marital quality and CVD risk measure quality at a single point^8^ and are unable to examine the importance of temporal changes in relationship quality. Only two studies have investigated the effect of longitudinal changes in relationship quality on outcomes related to CVD risk.^13 14^ One studied women (n=413) finding those in higher quality marriages was found to have lower risk of developing the metabolic syndrome.^14^ Another studied effects of relationship quality on dichotomous measures of CVD risk were such as hypertension, heart rate >80 bpm or C-reactive protein >3.0 mg/L in women (n=459) and men (n=739).^13^ This study suggested some effects on women but not men and few associations persisted after adjustment for confounders.

The present study uses 19 years of data from the Avon Longitudinal Study of Parents and Children (ALSPAC) to assess the association between longitudinal marital relationship quality and a range of CVD risk factors in married men. Men were the focus of this study as they have greater CVD risk than women in middle age,^15^ and display detectable risk factor differences earlier. We predicted that if marital quality was important for CVD risk, we would observe better CVD risk profiles in those with consistently better relationships, and changes in relationship quality over time (either getting worse or better) would be associated with similar changes in risk factor profiles after a sufficient latency period.

## Methodology

### Study population

Subjects were married men from the ALSPAC cohort. Details of ALSPAC have been published previously,^16^ and a searchable data dictionary is available online (www.bristol.ac.uk/alspac/). The study enrolled 14 541 pregnant women from in and around Bristol (UK) with estimated delivery dates between 1 April 1991 and 31 December 1992. There were 14 062 live births, and the children, the mothers and the mothers’ partners (referred to as ‘male partners’) have been studied to the present day. The male partners’ data are the focus of this paper. The baseline (T1) sample is restricted to men who were married, reported being the father of the ALSPAC child, and had T1 relationship and covariate data. Male partners were excluded from the final analysis if they were lost to follow-up, lacked complete outcome, exposure or covariate data, or were not married and confirmed as the study child’s biological father at 6.4 years (T2) and 18.8 years (T3) after T1. Ethical approval was obtained from ALSPAC Ethics and Law Committee, Local Research Ethics Committees and National Health Service National Research Ethics Service Committee (North West – Haydock). Full references for ALSPAC study ethics approval are available online.^17^

### Exposure

**Figure 1:**
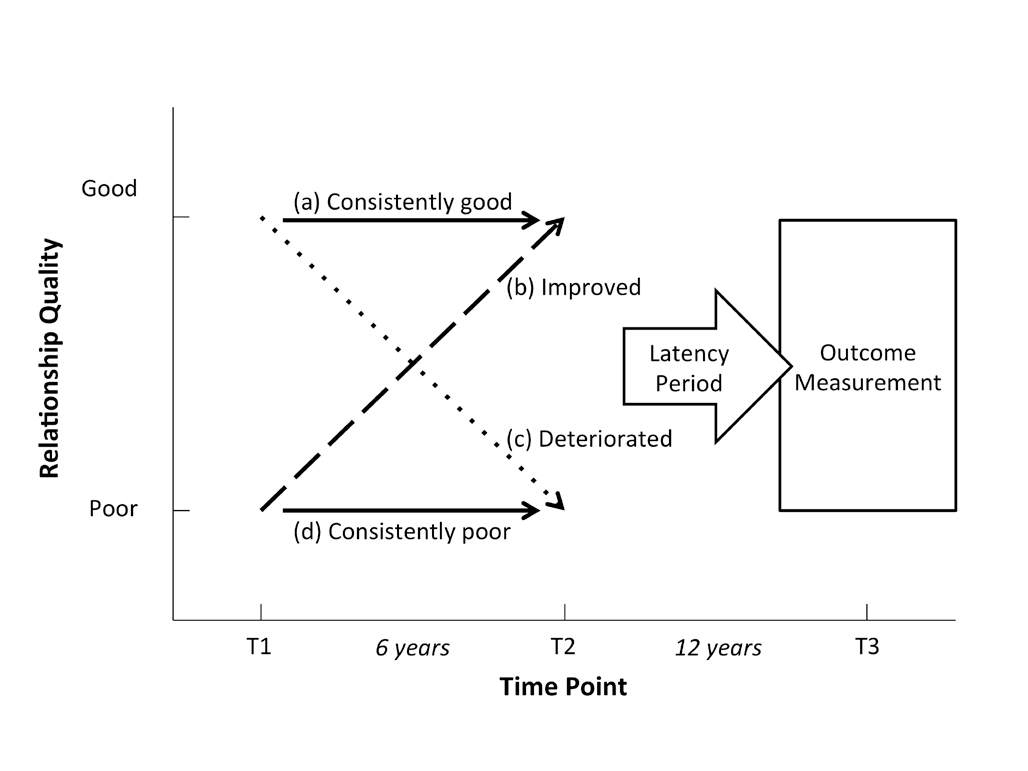
Longitudinal patterns of marital quality and outcome measurements.

Longitudinal relationship quality was measured using the 12-item ‘care’ subscale of the ‘measure of intimate bonds’ scale^18^ at T1 when the men had a mean age of 34 years and 6.4 years later at T2. We chose not to use the ‘control’ subscale of this instrument, as conceptually it was unclear whether relationships with high or low control were better or worse. The care subscale uses a Likert scale from 0 to 3 to rate agreement with 12 statements regarding relationship quality, a higher score indicates a better relationship. Due to marked skewness, we grouped data into tertiles and categorised a good relationship as being in tertiles 2 or 3, and a poor relationship as tertile 1. As we had repeat data at two time points (T1, T2), we derived the following four patterns (figure 1): (a) consistently good (good at T1 and T2), (b) improving relationships (poor at T1 and good at T2), (c) deteriorating relationships (good at T1 and poor at T2), (d) consistently poor relationships (poor at T1 and T2).

### Main outcome measures

We hypothesised that there would be a latency period between changes in marital quality and CVD risk factors so these were measured in the subsequent follow-up period (T3), 18.8 years after T1 and during a 2-hour ALSPAC clinic session between September 2011 and February 2013. Fasting blood samples (minimum 8 hours) were analysed for total cholesterol, high-density lipoprotein (HDL) cholesterol, low-density lipoprotein (LDL) cholesterol, triglycerides and fasting glucose, all measured in mmol/L. Resting heart rate, recorded in beats/min, and mean seated systolic blood pressure (BP) and diastolic BP, recorded in mm Hg, were measured by two readings from a single arm using an Omron M6 BP/Pulse monitor. Body mass index (BMI) was calculated at T3 (weight (kg)/height (m)^2^); weight was measured with Tanita scales (TBF401-A) and recorded to the nearest 0.1 kg, and height was measured to the nearest 1 mm using a Harpenden stadiometer.

### Confounders and intermediaries

Potential confounders were age measured at T1, height measured objectively at T3 (short stature is associated with increased CVD risk)^19^ and adult socioeconomic position (SEP). Three measures of self-reported SEP were included: housing tenure 3 years after T1, highest educational qualification 3 years prior to T1 and financial difficulties with common expenditures (range 0–40 with higher score indicating worse outcome) at T1 (categorised in quartiles). We did not adjust for baseline smoking and alcohol in our main models for the following reasons. Alcohol consumption could be both a confounder and intermediary as heavy drinking may lead to worse marital quality but similarly worse marital quality may lead to heavier drinking; hence adjustment may underestimate the association. While smoking is associated with other CVD risk factors, it was not clear how it would be itself determined by marital quality other than via its association with socioeconomic status, which has been adjusted for. A sensitivity analysis adjusting for smoking and alcohol was undertaken to evidence our approach. Smoking was quantified using self-report of number of cigarettes smoked a day at T1 (none, 1–9, 10–19, 20+). Alcohol consumption was measured by self-reported approximate unit consumption at T1.

We hypothesised that a poor relationship may lead to a less healthy diet and/or less physical activity with subsequent increased obesity, so BMI (measured at T3) was identified as the main potential mediator. To examine how BMI at T3 may have changed from T1, we used self-reported height and weight prior to T1 as a proxy for T1 BMI.

### Statistical methods

Baseline characteristics of the selected and excluded sample were analysed using Χ^2^ tests for discrete, and t-tests for continuous variables. Associations between both T1 relationship quality and longitudinal relationship quality patterns with CVD risk factor outcomes were analysed using regression models adjusting for: (1) age, (2) age and confounders, and (3) age, confounders and intermediaries. For the BMI outcome, we examined associations with CVD risk factors both without and with adjustment for BMI prior to T1, the latter allowing assessment of relative change in BMI over the study period.

### Sensitivity analyses

We used multiple imputation using chained equations in post-hoc sensitivity analysis of the association between T1 relationship quality and CVS risk factor outcomes, whereby missing T1 exposure and covariate data were imputed for the sub-group of participants with complete outcome data at T3 (50 datasets imputed). As some of the men in the study reported that they had treated hypertension we repeated the BP analysis but added 10 and 5 mm Hg to the observed systolic and diastolic pressures respectively to approximate underlying BP without treatment^20 21^ for the regression analyses of longitudinal relationship quality and CVD risk factor outcomes. We also repeated our main analyses adjusting for marital quality at T3 to account for changes in relationship quality from T2 to T3, however, the temporal sequence between the risk factor and current marital quality is less clear as these were measured at the same time. Finally, we repeated our main analysis adjusting for smoking and alcohol consumption at T1.

## Results

**Figure 2:**
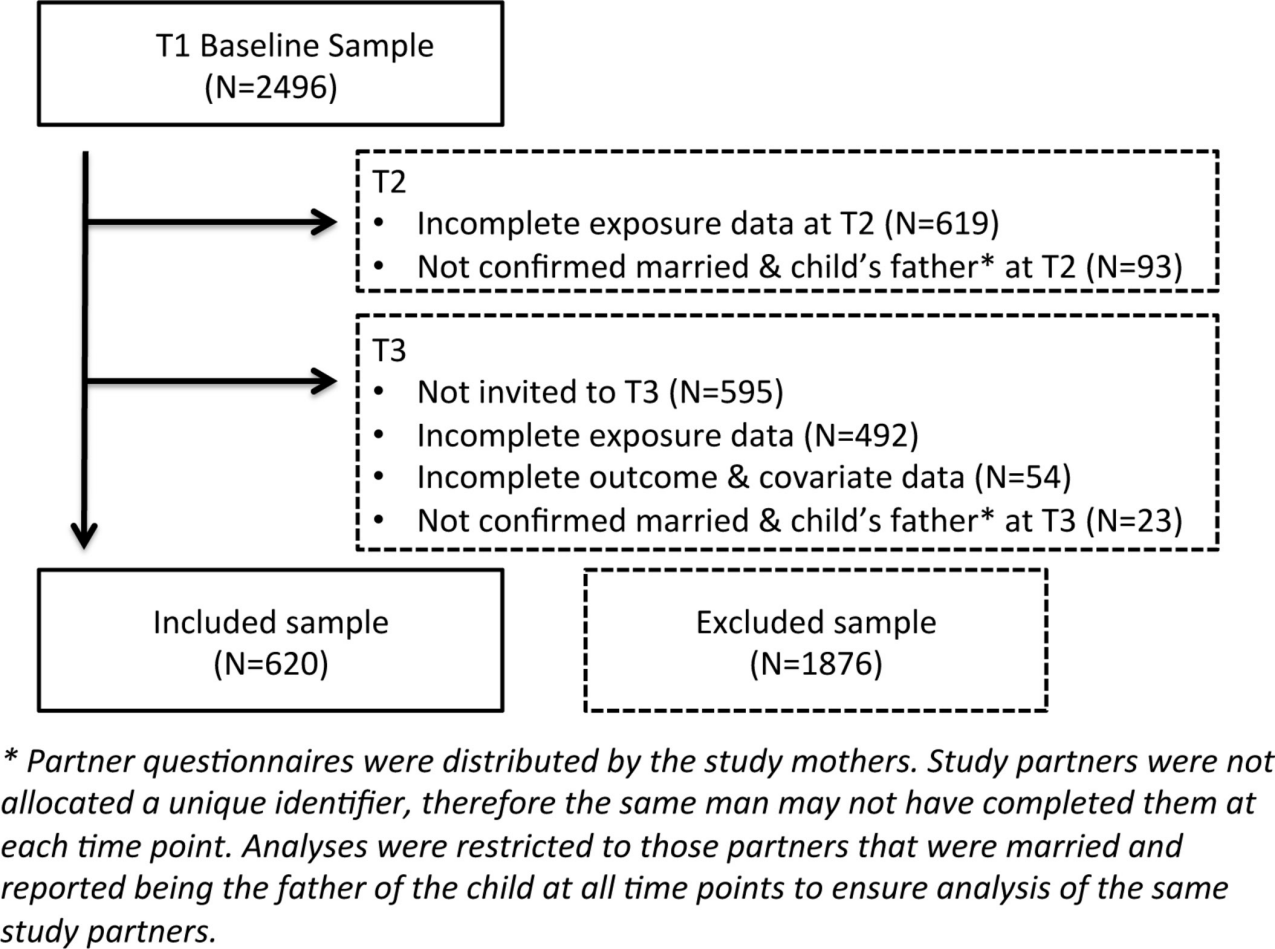
Participant eligibility, exclusions and complete case sample.

From our original sample of 2496, we had a complete case sample of 620 (25%) (figure 2). A large proportion of the ‘excluded’ sample was comprised those with incomplete T2 exposure data (25%), and those for whom a lack of contact details prevented invitation to the T3 clinic (24%). Prior to T3, communication with these men was via the index child’s mother. Men in the excluded sample were slightly younger, had greater adiposity, were of lower educational level, had more financial difficulties and worse marital quality (table 1). Relationship quality at T1 (being in a poor rather than good relationship) was a predictor of divorce or separation at T3 (11.9% vs 4.75%, p=0.002). There was no association (p=0.767) between levels of ‘control’ in a relationship at T1 and relationship status (divorce/separation and so on) at T3, evidencing the methodological decision to use the ‘care’ subscale of the ‘measure of intimate bonds’^18^ to represent relationship quality.

**Table 1:** Baseline covariate comparison of included and excluded sample at T1

Baseline data from T1	Included sample (n=620)	Excluded sample (n=1876)	p Value
Mean age (SD) (years)	35.9 (5.07)	34.7 (5.37)	<0.001 *
Mean height † (SD) (cm)	177 (6.19)	177 (6.79)	0.76 *
Mean BMI † (SD) (kg/m^2^)	24.6 (2.84)	25.2 (3.31)	<0.001*
Housing tenure‡ (%)			
Own property and no mortgage	4.68	3.78	0.03§
Being bought/mortgaged	90.2	88.2	
Private Rented	2.74	2.51	
Council/Housing Assoc. rented	1.29	3.57	
Other	1.13	1.92	
Financial difficulties (%)			
Least difficulty	51.0	44.8	0.007 §
Slight difficulty	15.5	14.3	
Moderate difficulty	20.0	22.4	
Most difficulty	13.6	18.5	
Highest educational qualification † (%)			
*≥*Degree	41.6	28.7	<0.001 §
A Level	29.4	30.0	
O Level	18.7	22.6	
Vocational	3.55	6.93	
CSE	6.77	11.8	
Number of cigarettes a day (%)			
None	87.7	78.5	<0.001 §
1–9	5.16	9.28	
10–19	4.84	7.30	
20+	2.26	4.90	
Alcohol consumption (%)			
Less than once a week	26.3	28.6	0.030 §
At least once a week	45.0	43.8	
1–2 glasses near every day	24.2	20.5	
≥3 glasses nearly every day	4.52	7.14	
Relationship quality score (%)			
Least caring	29.8	33.6	0.015 §
Moderately caring	32.1	34.6	
Most caring	38.1	31.8	

* t-test.

†Data from 3 years prior to T1 as approximation to T1.

‡Data from 3 years after to T1 as approximation to T1.

§ Χ^2^ test.

We found few differences in the age-adjusted CVD risk factor outcomes at T3 when comparing good and poor relationship at T1 (table 2) except for LDL cholesterol, which was paradoxically lower for those in poor relationships, and the change in BMI from T1 where those in poor relationships were less likely to put on weight (table 2 Model 1). Conditioning for confounders made little difference to these estimates (table 2 Model 2). Post-hoc sensitivity analysis through multiple imputation using chained equations (n=620 in original complete case versus n=876 in imputed sample) led to a slight attenuation of the observed relationship between T1 relationship quality and change in BMI measured at T3 (p=0.008 to 0.02) and T3 LDL (p=0.05 to 0.08), other associations were unchanged.

**Table 2:** T1 relationship quality and T3 CVD risk factor outcomes

	Mean (95% CI)	Mean difference compared with good relationship (95% CI)	
CVD risk factor outcome	Good relationship (n=435)	Poor relationship (n=185)	
Cardiovascular	Age adjusted	Age adjusted	Age plus confounder
Systolic BP (mm Hg)	132.81 (131.53 to 134.09)	−0.23 (−2.50 to 2.04)	−0.46 (−2.74 to 1.82)
Diastolic BP (mm Hg)	77.81 (76.95 to 78.67)	−0.48 (−2.01 to 1.05)	−0.70 (−2.24 to 0.83)
Resting heart rate (bpm)	65.22 (64.23 to 66.20)	−0.85 (−2.59 to 0.89)	−0.91 (−2.66 to 0.84)
Anthropometric			
BMI (kg/m^2^)	27.46 (27.11 to 27.81)	−0.22 (−0.84 to 0.40)	−0.33 (−0.94 to 0.29)
∆BMI (kg/m^2^)	4.81 (3.10 to 6.51)	−0.51 (−0.93 to −0.08)*	−0.57
Lipids			
Total cholesterol (mmol/L)	5.18 (5.10 to 5.27)	−0.11 (−0.27 to 0.05)	−0.12 (−0.27 to 0.04)
HDL (mmol/L)	1.29 (1.27 to 1.32)	0.00 (−0.04 to 0.04)	0.01 (−0.04 to 0.05)
LDL (mmol/L)	3.25 (3.17 to 3.33)	−0.14 (−0.28 to −0.00)*	−0.14 (−0.28 to −0.00)*
Triglycerides (mmol/L)	1.40 (1.35 to 1.46)	−0.03 (−0.13 to 0.08)	−0.04 (−0.14 to 0.07)
Glucose (mmol/L)	5.65 (5.54 to 5.76)	0.11 (−0.08 to 0.31)	0.09 (−0.11 to 0.28)

Regression Model 1: outcome adjusted for age.

Regression Model 2: outcome adjusted for age +confounders (housing tenure +financial difficulties+highest educational qualification +height).

*p = 0.01–0.05 **p=0.001–0.01 ***p<0.001.

BMI, body mass index; BP, blood pressure; CVD, cardiovascular disease; HDL, high-density lipoprotein; LDL, low-density lipoprotein.

We found that men with persistent poor-quality relationships had very small adverse differences in their CVD risk factors compared with those with persistently good relationships but these were all consistent with chance sampling variability (table 3). Clearer patterns emerged for those with either improving or deteriorating marital quality. Both systolic and diastolic BPs went down or up in line with improving or deteriorating marital quality compared with those with persistent good relationships, though only the increase in diastolic BP (2.74 mm Hg, 95% CI 0.50 to 4.98) was unlikely to be due to chance. Those with improving relationships also showed lower total cholesterol mainly through effects on LDL cholesterol (−0.25 mmol/L, 95% CI −0.46 to −0.03). Change in both these risk factors for the improving quality group may have been driven by weight loss (change in BMI −1.07 kg/m^2^, 95% CI −1.73 to −0.42 kg/m^2^). Weaker effects on increasing BMI were seen for the deteriorating marital quality group (change in BMI 0.53 kg/m^2^, 95% CI −0.09 to 1.15 kg/m^2^ p=0.09). When we repeated our analysis conditioning on T3 BMI as a potential intermediary (online supplementary etable 1), we found minimal differences in the association with diastolic BP (p=0.04, rather than 0.02) and LDL cholesterol (p=0.02, rather than 0.03).

10.1136/jech-2017-209178.supp1 Supplementary file 1

**Table 3:** Relationship trajectory and T3 CVD risk factor outcomes

	Mean (95% CI)	Mean difference compared with good relationship (95% CI)					
CVD risk factor outcome	Good relationship (n=362)	Improving relationship (n=65)		Deteriorating relationship (n=73)		Poor relationship (n=120)	
Cardiovascular	Age adjusted	Age adjusted	Age+confounders	Age adjusted	Age+confounders	Age adjusted	Age+confounders
Systolic BP (mm Hg)	132.63 (131.24 to 134.02)	−2.72 (−6.20 to 0.75)	−2.75 (−6.26 to 0.75)	1.06 (−2.25 to 4.37)	1.42 (−1.92 to 4.76)	1.39 (−1.32 to 4.11)	1.14 (−1.59 to 3.88)
Diastolic BP (mm Hg)	77.37 (76.44 to 78.30)	−2.05 (−4.38 to 0.28)	−2.24 (−4.59 to 0.11)	2.64 (0.42 to 4.86)*	2.74 (0.50 to 4.98)*	1.05 (−0.77 to 2.87)	0.83 (−1.00 to 2.66)
Resting heart rate (bpm)	64.93 (63.86 to 65.99)	−2.49 (−5.15 to 0.18)	−2.39 (−5.07 to 0.30)	1.74 (−0.80 to 4.27)	1.62 (−0.94 to 4.18)	0.48 (−1.60 to 2.56)	0.30 (−1.80 to 2.40)
Anthropometric							
BMI (kg/m^2^)	27.36 (26.98 to 27.74)	−0.45 (−1.40 to 0.50)	−0.57 (−1.51 to 0.37)	0.62 (−0.29 to 1.52)	0.75 (−0.16 to 1.65)	0.06 (−0.68 to 0.81)	−0.01 (−0.74 to 0.73)
∆BMI (kg/m^2^)	4.67 (2.98 to 6.37)	−1.02 (−1.67 to −0.37)**	−1.07 (−1.73 to −0.42)**	0.46 (−0.16 to 1.08)	0.53 (−0.09 to 1.15)	−0.11 (−0.62 to 0.40)	−0.16 (−0.67 to 0.34)
Lipids							
Total cholesterol (mmol/L)	5.18 (5.08 to 5.27)	−0.24 (−0.48 to −0.00)*	−0.24 (−0.48 to 0.01)	0.05 (−0.18 to 0.28)	0.05 (−0.18 to 0.29)	−0.02 (−0.21 to 0.16)	−0.04 (−0.23 to 0.15)
HDL (mmol/L)	1.29 (1.26 to 1.32)	0.01 (−0.05 to 0.08)	0.03 (−0.04 to 0.09)	0.00 (−0.06 to 0.07)	0.00 (−0.06 to 0.07)	−0.01 (−0.06 to 0.05)	−0.00 (−0.06 to 0.05)
LDL (mmol/L)	3.26 (3.17 to 3.34)	−0.26 (−0.47 to −0.04)*	−0.25 (−0.46 to −0.03)*	−0.04 (−0.25 to 0.16)	−0.04 (−0.24 to 0.17)	−0.09 (−0.25 to 0.08)	−0.09 (−0.26 to 0.07)
Triglycerides (mmol/L)	1.39 (1.33 to 1.45)	−0.11 (−0.27 to 0.05)	−0.12 (−0.29 to 0.04)	0.09 (−0.06 to 0.24)	0.10 (−0.06 to 0.25)	0.04 (−0.08 to 0.17)	0.03 (−0.09 to 0.16)
Glucose (mmol/L)	5.61 (5.49 to 5.73)	0.21 (−0.09 to 0.51)	0.19 (−0.11 to 0.49)	0.23 (−0.05 to 0.51)	0.23 (−0.05 to 0.52)	0.12 (−0.12 to 0.35)	0.09 (−0.14 to 0.33)

Regression model 1: outcome adjusted for age.

Regression model 2: outcome adjusted for age +confounders (housing tenure +financial difficulties+highest educational qualification +height).

*p = 0.01–0.05 **p=0.001– 0.01 ***p<0.001.

BMI, body mass index; BP, blood pressure; CVD, cardiovascular disease; HDL, high-density lipoprotein; LDL, low-density lipoprotein.

Sensitivity analyses accounting for the effects of medically treated BP (online supplementary etable 2) increased the mean effect size in the deteriorating relationship group on diastolic BP (2.95 mm Hg, 95% CI 0.53 to 5.36 mm Hg) though it slightly weakened the effect for the improving quality group. Further adjustment for marital quality at T3 (online supplementary etable 3) made little difference to the association between relationship quality and diastolic BP or change in BMI. However, accounting for T3 relationship quality did attenuate the association between improving relationship quality and LDL (p=0.09, rather than p=0.03). Further sensitivity analysis adjusting for smoking and alcohol consumption made little difference to the results from the main confounder model (online supplementary etable 4).

## Discussion

Married men have been shown to have reduced CVD mortality and morbidity, though the reasons for this remain unclear. This is the first study to assess the association between repeated measures of marital relationship quality and a broad range of CVD risk factors over two decades. Overall, among married men there were only very modest benefits in terms of CVD risk factors, if at all, when comparing men in persistently good or bad relationships. However, changes in marital quality had positive or negative effects on lipids and BP, as we had predicted a priori, and a modest degree of this may have been mediated through changes in adiposity (online supplementary etable 1). These findings were little changed after adjustment for measures of socioeconomic status. Traditionally, beneficial effects of marital status were thought to be mediated by either health selection, confounding by socioeconomic status, or psychosocial mechanisms. The latter argument has been used to support the observation that men appear to gain more benefit than women, as women have larger social networks and are less dependent on their partner than men.^22^ An alternative explanation relates to shared environment. Data from the English Longitudinal Study of Ageing (ELSA) found that men and women are more likely to make a positive health behaviour change if their partner also changes their behaviour.^10^ It seems reasonable to assume that this effect might be modified by the quality of the relationship. This is consistent with our findings for changes in BMI which are known to have greater effects on altering BP than lipids as shown by the Look AHEAD randomised controlled trial.^23^ The similarity of CVD risk factors for men in persistently good and bad marriages suggests a number of possibilities; that quality of marital relationship is unimportant; that there could be some habituation after a period of time—so the emotional effects of marital quality are no longer salient or reporting bias. Interestingly, the UK million women study failed to find any association between happiness and ischaemic heart disease mortality after appropriate adjustment, suggesting that a simplistic psychosocial explanation may be inadequate.^24^ Alternatively, reporting marital status at one time point, rather than many as in the present study, may be a better measure of a personality trait (‘glass half empty or half full’) rather than marital quality per se, which is better captured by relative changes over time. If personality trait is itself unrelated to CVD risk factors, then this would mask true differences by marital quality.

There are only two comparative studies. Troxel *et al*^14^ measured relationship quality at baseline and after 3 years in 321 women from the Pittsburgh Healthy Women Study. They reported presence or absence of ‘metabolic syndrome’, at approximately 11.5 years, finding women in dissatisfying marriages to be at higher risk. They measured three relationship trajectories, representing (a) consistent satisfaction, (b) consistent dissatisfaction, and (c) change in marital satisfaction in either direction (rather than the direction of change), and hence it is not possible to compare their findings with those of the present study. A more recent paper by Liu and Waite^13^ also investigated the effect of longitudinal relationship quality on binary measures of CVD risk using outcomes of the presence or the absence of hypertension (both measured or self reported), ‘rapid heart rate’ defined as a heart rate >80 bpm and C-reactive protein >3 mg/L over 5 years of follow-up in a cohort of 739 married men and 459 women. For women, they found that worsening relationships were associated with more adverse CVD risk factors though the effects were more marked at older ages. For men their results were weak and inconsistent from which they argued that men may be less likely to internalise a poor relationship than women.

The breadth of data available through the ALSPAC study enabled us to examine marital quality data at three time points over 19 years and test associations with CVD risk factors, while adjusting for several measures of socioeconomic status. However, there has been participant attrition over the course of the study with likely loss to follow-up bias and a consequent reduction of power. As expected, men who were excluded from the analysis were more likely to be poorer, drink more alcohol and have worse marital quality at T1. Wives in poor relationships may have been less likely to ask their partners to attend the follow-up clinic and hence would be missing. Those whose marriage dissolved prior to or during the study were excluded. These biases would systematically attenuate any true relationship so that our observed findings may underestimate the true effect. However, our imputation analyses resulted in a modest attenuation of effects suggesting that men with missing covariates who had outcomes at T3 had weaker associations with marital quality. As our study looked at men, these findings may not be generalizable to women. Our exposure measure of marital quality is one of many available^8^ and may not capture all aspects of relationships, though did have predictive validity in terms of future divorce risk. We tested multiple CVD risk factors so it is possible that some of the associations are type I errors.

We used a question on marital status as a marker of a long-term partnership, though it is unclear whether the same effects would have been seen with cohabitation or whether marriage has additional effects. This is important as marriage and cohabitation trends in developed countries have changed substantially in the last 50 years, with cohabiting in the UK doubling between 1996 and 2012.^25^

Changes in the quality of a marital relationship appear to predict CVD risk, though consistently good or poor relationship groups were not very different. At this stage, it is unclear whether these patterns will be reflected in actual rates of disease onset as the cohort is still relatively young. Assuming a causal association, then marriage counselling for couples with deteriorating relationships may have added benefits in terms of physical health over and above psychological well-being, though in some cases ending the relationship may be the best outcome.

**What is already known on this subject**

- Associations between marital status and cardiovascular disease (CVD) morbidity have long been observed in cohort studies, however it is debated whether this is causal or due to selection bias.
- Analysing effects of changes in relationship quality within marriages controls for selection, providing stronger evidence to assess for an association.
- Only two small studies have investigated the association between changes in marital relationship quality and CVD-related risk factor outcomes, with unclear findings due to difficulty measuring longitudinal changes in relationship quality and binary representations of CVD risk outcome measures.

**What this study adds**

- Improvement and deterioration of longitudinal relationship quality appears to be associated with respectively positive and negative associations with a broad range of objectively measured cardiovascular disease risk factors.
- Further research needs to determine if effective marriage counselling, or when appropriate, abandoning a deteriorating relationship, has longer term physical health benefits over and above psychological well-being.
