# Supplementary material for: Changes in marital quality over 6 years and its association with cardiovascular disease risk factors in men: findings from the ALSPAC prospective cohort study
Source: J Epidemiol Community Health. 2017 Oct 9;71(11):1094–100. doi: 10.1136/jech-2017-209178 (PMC5847094; doi:10.1136/jech-2017-209178)
Supplement: Supplementary file 1 [file jech-2017-209178supp001.pdf]

eTable 1: Sensitivity analysis of relationship trajectory and T3 cardiovascular risk factor outcomes adjusting for age, confounders and intermediaries

	**Mean (95% CI)**	**Mean difference compared to Good Relationship (95% CI)**		
**CVS Risk Factor Outcome**	**Good Relationship (n=362)**	**Improving relationship (n=65)**	**Deteriorating relationship (n=73)**	**Poor Relationship (n=120)**
**Cardiovascular**				
Systolic BP (mmHg)	130.48 (125.36 to 135.59)	-2.31 (-5.74 to 1.13)	0.83 (-2.44 to 4.11)	1.15 (-1.53 to 3.82)
Diastolic BP (mmHg)	74.40 (70.97 to 77.83)	-1.95 (-4.25 to 0.35)	2.36 (0.16 to 4.56)*	0.83 (-0.96 to 2.63)
Resting heart rate (bpm)	64.62 (60.71 to 68.53)	-2.02 (-4.64 to 0.60)	1.14 (-1.37 to 3.64)	0.30 (-1.74 to 2.35)
**Lipids**				
Total Cholesterol (mmol/l)	4.90 (4.54 to 5.26)	-0.24 (-0.48 to 0.01)	0.06 (-0.18 to 0.29)	-0.04 (-0.23 to 0.15)
HDL (mmol/l)	1.33 (1.23 to 1.42)	0.01 (-0.05 to 0.08)	0.02 (-0.04 to 0.08)	-0.01 (-0.05 to 0.04)
LDL (mmol/l)	3.09 (2.77 to 3.41)	-0.25 (-0.46 to -0.03)*	-0.04 (-0.24 to 0.17)	-0.09 (-0.26 to 0.07)
Triglycerides (mmol/l)	1.30 (1.07 to 1.52)	-0.09 (-0.24 to 0.06)	0.05 (-0.09 to 0.20)	0.03 (-0.09 to 0.15)
Glucose (mmol/l)	5.28 (4.84 to 5.72)	0.22 (-0.07 to 0.52)	0.19 (-0.09 to 0.47)	0.09 (-0.13 to 0.32)

Regression Model: Outcome adjusted for age + confounders (housing tenure + financial difficulties +highest educational qualification + height) + intermediaries (BMI at T3)

* p = 0.01 to 0.05 ** p = 0.001 to 0.01 *** p < 0.001

eTable 2: Sensitivity analysis of SBP and DBP adjusting for antihypertensive treatment

	Mean (95% CI)	Mean difference compared to Good Relationship (95% CI)					
**CVS Risk Factor Outcome**	**Good Relationship (n=362)**	**Improving relationship (n=65)**		**Deteriorating relationship (n=73)**		**Poor Relationship (n=120)**	
**Cardiovascular**	**Age adjusted**	**Age adjusted**	**Age + confounders**	**Age adjusted**	**Age + confounders**	**Age adjusted**	**Age + confounders**
Adjusted[b] Systolic BP (mmHg)	134.18 (132.61 to 135.74)	-2.42 (-6.33 to 1.50)	-2.52 (-6.47 to 1.43)	1.42 (-2.30 to 5.15)	1.83 (-1.94 to 5.61)	1.37 (-1.70 to 4.43)	1.10 (-1.99 to 4.19)
Adjusted[c] Diastolic BP (mmHg)	78.14 (77.14 to 79.15)	-1.89 (-4.40 to 0.62)	-2.12 (-4.65 to 0.41)	2.82 (0.43 to 5.21)*	2.95 (0.53 to 5.36)*	1.03 (-0.93 to 3.00)	0.81 (-1.17 to 2.79)

Regression Model 1: Outcome adjusted for age
Regression Model 2: Outcome adjusted for age + confounders (housing tenure + financial difficulties + highest educational qualification + height)
[b] Systolic BP adjusted by +10 mmHg as sensitivity analysis to account for antihypertensive treatment
[c] Diastolic BP adjusted by +5 mmHg as sensitivity analysis to account for antihypertensive treatment
* p = 0.01 to 0.05 ** p = 0.001 to 0.01 *** p < 0.001

eTable 3: Sensitivity analysis of relationship trajectory and T3 cardiovascular risk factor outcomes adjusting relationship quality at T3

	Mean (95% CI)	Mean difference compared to Good Relationship (95% CI)					
**CVS Risk Factor Outcome**	**Good Relationship (n=362)**	**Improving relationship (n=65)**		**Deteriorating relationship (n=73)**		**Poor Relationship (n=120)**	
**Cardiovascular**	**Age adjusted**	**Age adjusted**	**Age + confounders**	**Age adjusted**	**Age + confounders**	**Age adjusted**	**Age + confounders**
Systolic BP (mmHg)	132.02 (129.02 to 135.03)	-2.42 (-6.00 to 1.15)	-2.49 (-6.10 to 1.11)	1.39 (-2.09 to 4.87)	1.67 (-1.84 to 5.18)	1.88 (-1.31 to 5.07)	1.51 (-1.70 to 4.73)
Diastolic BP (mmHg)	76.89 (74.88 to 78.91)	-1.81 (-4.21 to 0.58)	-2.01 (-4.43 to 0.40)	2.90 (0.57 to 5.23)*	2.98 (0.63 to 5.33)*	1.43 (-0.71 to 3.57)	1.20 (-0.96 to 3.35)
Resting heart rate (bpm)	65.26 (62.95 to 67.57)	-2.37 (-5.11 to 0.37)	-2.23 (-4.99 to 0.54)	1.70 (-0.97 to 4.37)	1.63 (-1.06 to 4.32)	0.38 (-2.07 to 2.82)	0.27 (-2.20 to 2.73)
**Anthropometric**							
BMI (kg/m^2)	27.69 (26.87 to 28.52)	-0.48 (-1.46 to 0.50)	-0.62 (-1.59 to 0.35)	0.51 (-0.45 to 1.46)	0.63 (-0.31 to 1.58)	-0.12 (-1.00 to 0.75)	-0.19 (-1.06 to 0.67)
ΔBMI (kg/m^2)	4.88 (3.11 to 6.66)	-0.99 (-1.66 to -0.33)**	-1.06 (-1.73 to -0.39)**	0.41 (-0.24 to 1.06)	0.49 (-0.16 to 1.14)	-0.21 (-0.80 to 0.39)	-0.25 (-0.84 to 0.35)
**Lipids**							
Total Cholesterol (mmol/l)	5.10 (4.89 to 5.31)	-0.21 (-0.46 to 0.04)	-0.21 (-0.45 to 0.04)	0.09 (-0.16 to 0.33)	0.09 (-0.15 to 0.33)	0.03 (-0.19 to 0.25)	0.02 (-0.20 to 0.24)
HDL (mmol/l)	1.30 (1.24 to 1.35)	0.01 (-0.06 to 0.08)	0.03 (-0.04 to 0.10)	0.00 (-0.07 to 0.07)	0.00 (-0.06 to 0.07)	-0.01 (-0.07 to 0.05)	-0.01 (-0.07 to 0.05)
LDL (mmol/l)	3.15 (2.96 to 3.33)	-0.20 (-0.42 to 0.02)	-0.19 (-0.41 to 0.03)	0.02 (-0.20 to 0.23)	0.02 (-0.20 to 0.23)	0.00 (-0.19 to 0.20)	-0.01 (-0.21 to 0.19)
Triglycerides (mmol/l)	1.35 (1.21 to 1.49)	-0.11 (-0.27 to 0.06)	-0.12 (-0.29 to 0.04)	0.10 (-0.06 to 0.26)	0.11 (-0.05 to 0.27)	0.06 (-0.08 to 0.21)	0.06 (-0.09 to 0.21)
Glucose (mmol/l)	5.62 (5.36 to 5.88)	0.21 (-0.10 to 0.51)	0.18 (-0.12 to 0.49)	0.22 (-0.07 to 0.52)	0.23 (-0.06 to 0.53)	0.11 (-0.16 to 0.38)	0.10 (-0.18 to 0.37)

Regression Model 1: Outcome adjusted for age + relationship quality at T3
Regression Model 2: Outcome adjusted for age + confounders (housing tenure + financial difficulties+highest educational qualification+height) + T3 relationship quality
* p = 0.01 to 0.05 ** p = 0.001 to 0.01 *** p < 0.001

eTable 4: Sensitivity analysis of relationship trajectory and cardiovascular risk factor outcomes adjusting for age, confounders, smoking and alcohol consumption

	Mean (95% CI)	Mean difference compared to Good Relationship (95% CI)		
**CVS Risk Factor Outcome**	**Good Relationship (n=362)**	**Improving relationship (n=65)**	**Deteriorating relationship (n=73)**	**Poor Relationship (n=120)**
**Cardiovascular**				
Systolic BP (mmHg)	127.29 (121.85 to 132.73)	-2.55 (-6.06 to 0.97)	1.71 (-1.66 to 5.08)	1.16 (-1.61 to 3.93)
Diastolic BP (mmHg)	72.43 (68.80 to 76.07)	-2.21 (-4.55 to 0.14)	2.86 (0.60 to 5.11)*	0.76 (-1.10 to 2.61)
Resting heart rate (bpm)	64.46 (60.33 to 68.60)	-2.57 (-5.25 to 0.10)	1.44 (-1.13 to 4.00)	-0.36 (-2.47 to 1.74)
**Anthropometric**				
BMI (kg/m^2)	25.44 (23.96 to 26.91)	-0.60 (-1.55 to 0.35)	0.71 (-0.20 to 1.62)	-0.05 (-0.80 to 0.70)
ΔBMI (kg/m^2)	4.16 (2.25 to 6.07)	-1.10 (-1.75 to -0.44)**	0.50 (-0.13 to 1.12)	-0.19 (-0.71 to 0.33)
**Lipids**				
Total Cholesterol (mmol/l)	4.77 (4.39 to 5.14)	-0.24 (-0.49 to 0.00)*	0.02 (-0.21 to 0.26)	-0.04 (-0.23 to 0.15)
HDL (mmol/l)	1.32 (1.22 to 1.43)	0.03 (-0.03 to 0.10)	0.01 (-0.06 to 0.07)	-0.01 (-0.06 to 0.04)
LDL (mmol/l)	3.05 (2.71 to 3.38)	-0.25 (-0.47 to -0.04)*	-0.05 (-0.26 to 0.15)	-0.10 (-0.27 to 0.07)
Triglycerides (mmol/l)	1.17 (0.92 to 1.42)	-0.13 (-0.29 to 0.03)	0.07 (-0.08 to 0.23)	0.03 (-0.10 to 0.16)
Glucose (mmol/l)	4.99 (4.53 to 5.45)	0.21 (-0.09 to 0.51)	0.25 (-0.03 to 0.54)	0.15 (-0.08 to 0.39)

Regression Model: Outcome adjusted for age + confounders (housing tenure + financial difficulties +highest educational qualification + height) + smoking and alcohol consumption at T1

* p = 0.01 to 0.05 ** p = 0.001 to 0.01 *** p < 0.001
